# Integrating single-cell RNA-seq and bulk RNA-seq to construct prognostic signatures to explore the role of glutamine metabolism in breast cancer

**DOI:** 10.3389/fendo.2023.1135297

**Published:** 2023-02-10

**Authors:** Shengbin Pei, Pengpeng Zhang, Huilin Chen, Shuhan Zhao, Yuhan Dai, Lili Yang, Yakun Kang, Mingjie Zheng, Yiqin Xia, Hui Xie

**Affiliations:** ^1^ Department of Breast Surgery, The First Affiliated Hospital of Nanjing Medical University, Nanjing, China; ^2^ Department of Thoracic Surgery, The First Affiliated Hospital of Nanjing Medical University, Nanjing, China

**Keywords:** SNX3, glutamine, metabolism, breast cancer, single-cell sequencing

## Abstract

**Background:**

Although breast cancer (BC) treatment has entered the era of precision therapy, the prognosis is good in the case of comprehensive multimodal treatment such as neoadjuvant, endocrine, and targeted therapy. However, due to its high heterogeneity, some patients still cannot benefit from conventional treatment and have poor survival prognoses. Amino acids and their metabolites affect tumor development, alter the tumor microenvironment, play an increasingly obvious role in immune response and regulation of immune cell function, and are involved in acquired and innate immune regulation; therefore, amino acid metabolism is receiving increasing attention.

**Methods:**

Based on public datasets, we carried out a comprehensive transcriptome and single-cell sequencing investigation. Then we used 2.5 Weighted Co-Expression Network Analysis (WGCNA) and Cox to evaluate glutamine metabolism-related genes (GRGs) in BC and constructed a prognostic model for BC patients. Finally, the expression and function of the signature key gene SNX3 were examined by *in vitro* experiments.

**Results:**

In this study, we constituted a risk signature to predict overall survival (OS) in BC patients by glutamine-related genes. According to our risk signature, BC patients can obtain a Prognostic Risk Signature (PRS), and the response to immunotherapy can be further stratified according to PRS. Compared with traditional clinicopathological features, PRS demonstrated robust prognostic power and accurate survival prediction. In addition, altered pathways and mutational patterns were analyzed in PRS subgroups. Our study sheds some light on the immune status of BC. In *in vitro* experiments, the knockdown of SNX3, an essential gene in the signature, resulted in a dramatic reduction in proliferation, invasion, and migration of MDA-MB-231 and MCF-7 cell lines.

**Conclusion:**

We established a brand-new PRS consisting of genes associated with glutamine metabolism. It expands unique ideas for the diagnosis, treatment, and prognosis of BC.

## 1 Introduction

The incidence of BC among women worldwide is exceptionally high, and it ranks first, according to recent reports (1). In the past few decades, the treatment strategy of BC has changed from the traditional radical mastectomy combined with radiotherapy and chemotherapy to a comprehensive multimodal treatment such as neoadjuvant chemotherapy, endocrine therapy, and targeted therapy combined with surgery (2). However, due to the high heterogeneity of BC, some patients still cannot benefit from endocrine therapy and targeted molecular therapy (3). Currently, breakthroughs in immune checkpoint antagonist therapy in other cancers have renewed interest in treating and preventing BC in the same way (4). However, only two drugs, palivizumab, and atezolizumab have received Food and Drug Administration (FDA) approval for immunotherapy in BC (5). Therefore, better prognostic tools and biomarkers that accurately predict and treat BC are urgently needed.

Metabolic reprogramming is a major feature of tumor cells (6–8). Glutamine and glutamate are non-essential amino acids, which are the main sources of nitrogen and carbon for the synthesis of amino acids, lipids, and nucleic acids, but are important for the metabolic processes of tumor cells (9). The conversion of glutamine to glutamate by glutaminase in the mitochondria is a key step (10). The most prevalent amino acid in plasma, glutamine is crucial for protein, nucleotide, and energy metabolism in mitochondria. Glutamine catabolism can provide large NADPH requirements for proliferating cells (11). Some tumor cells rely on glutamine for cell growth and activation of signaling molecules, such as mTOR kinase (12). Aggressive cancers such as triple-negative breast cancer (TNBC) avidly metabolize glutamine as a feature of their malignant phenotype (13). Targeting glutamine metabolism enhances responses to platinum-based chemotherapy in TNBC (14). Therefore, the development of glutamine-dependent cell growth or “glutamine addiction” is considered as a new target for tumor therapy. The use of genes related to glutamine metabolism to predict treatment efficacy and clinical prognosis warrants further investigation.

Single-cell RNA-seq (scRNA-seq) is a novel tool that allows for the genomic examination of individual cells in a population, allowing for the identification of uncommon cells linked with cancer and metastasis (15, 16). In the fields of lung cancer, breast cancer, liver cancer, and gastric cancer research, scRNA-seq studies have discovered different populations that may correlate with poor prognosis and medication resistance (17–20). Furthermore, this approach may be utilized to demonstrate the heterogeneity of the tumor microenvironment, with these subpopulations potentially serving as immunotherapeutic targets. Because of its capacity to distinguish cell subsets and biomarkers with possible treatments, scRNA-seq is also a promising technology that might assist in tailored therapy. In common complex diseases such as autoimmune diseases, neurodegenerative diseases, and respiratory diseases, single-cell maps reveal the presence of disease genes at relevant sites of specific cell subsets of the disease (21). In cancer research, risk signatures are frequently utilized to forecast prognostic outcomes. Li W et al. developed an osteosarcoma lung metastasis prediction model (22, 23), and these features were shown to be superior to conventional methods in predicting clinical prognosis. In the field of breast cancer research, the role of molecular regulation related to glutamine metabolism has not been fully revealed. Therefore, we included genes associated with glutamine metabolism in the construction of risk profiles to estimate novel strategies for predicting outcomes in BC patients.

In this study, we downloaded BC public data from the Cancer Genome Atlas (TCGA) and Gene Expression Omnibus (GEO) databases. Single-cell sequencing analysis was performed to find differential glutamine metabolism-related genes among individual BC cells. Using the Cox risk model and LASSO regression, new risk profiles were constructed based on the expression levels of genes related to glutamine metabolism in the TCGA-BC dataset (24). In addition, in breast cancer, glutamine metabolic profiles can be used to identify changes in immune infiltration and immune checkpoints. Our findings might offer fresh perspectives on the investigation of BC diagnosis and therapy.

## 2 Materials and methods

### 2.1 Transcriptome data acquired and processing

Breast cancer RNA expression profiles, gene mutation, and corresponding clinical data were retrieved from the TCGA database (n=1095) and divided into a training group and validation group by 6:4, in which the training group was used to construct the model, and the validation group was used to check the stability and accuracy of the model. Simultaneously, the GEO expression profiles of GSE20685 (n= 327) were downloaded for use as an external independent validation cohort. All data were in TPM format and log2 was transformed for subsequent analysis. Adjustments for the batch effect between TCGA-BC and GSE20685 were made with the “sva” package.

### 2.2 Single-cell sequencing data and glutamine-related genes acquired and processing

From the GEO database, the single-cell data set GSE161529 of BC was retrieved. There are ten samples in all in the dataset. We performed the quality control of scRNA-seq data by the “seurat” R package. We kept cells with less than 10% mitochondrial genes, cells with more than 200 genes overall, and genes whose expression spanned from 200 to 7000 and were expressed in at least three cells to keep high-quality scRNA-seq data. A total of 50,917 eligible cells were selected for further exploration. The remaining cells were further scaled and normalized using a linear regression model with the “Log-normalization” technique. After data normalization, the top 3,000 hypervariable genes were distinguished according to the “FindVariableFeatures” function. As these data were obtained from several samples, we utilized the “FindlntegrationAnchors” function of the canonical correlation analysis (CCA) method to eliminate the batch effects disrupting downstream analysis. Subsequently, we used the “IntegrateData” and “ScaleData” functions to properly integrate and scale the data, respectively. Cell type was annotated and then manually checked according to previous studies (25, 26). The GeneCards database served as a source for GRGs, and a total of 141 GRGs with a relevance score greater than 15.0 were selected for subsequent investigation.

### 2.3 AUCell

scRNA-seq data were used to obtain the most relevant genes affecting Glutamine metabolism (GM) activity. The “AUCell R” package, which determines the active status of gene sets in scRNA-seq data, was employed to assign GM activity scores to each cell lineage. The percentage of highly expressed gene sets in each cell was estimated using the gene expression rankings of each cell based on the area under the curve (AUC) value of the selected GRGs. AUC values were larger for cells that expressed more genes. Cells actively involved in GM gene sets were determined using the “AUCell explore Thresholds” function. The cells were then divided into high- and low-GM-AUC groups based on the median AUC score and visualized using the “ggplot2” R package.

### 2.4 Single sample gene set enrichment analysis

To calculate the precise score of a gene set enriched in a sample, ssGSEA analysis is frequently utilized (27). This study used ssGSEA analysis to determine the GM scores for each TCGA-BC patient.

### 2.5 Weighted co-expression network analysis

The “WGCNA” package in R implements WGCNA, a systems biology technique for creating the TCGA-BC gene co-expression network. WGCNA can be used to locate highly covarying gene sets and to pinpoint potential biomarker genes or therapeutic targets based on the connectivity of each gene set and the link between the gene set and the phenotype. In this work, WGCNA was used to identify the gene modules associated with GM score in BC and to identify the associated genes. Finally, module genes with the most remarkable correlation to glutamine score were selected for further analysis.

### 2.6 Establishment of a risk signature associated with glutamine

First, a univariate Cox analysis was used to extract the glutamine-related genes having prognostic value. Lasso regression was used to further screen prognostic GRGs and multivariate regression analysis was performed to further identify the model genes and risk coefficients. Each breast cancer can therefore be given a risk score using the algorithm in this manner. Patients in the TCGA-BC cohort can be split into high- and low-risk groups based on the median value. Then, we investigated how the two groups’ prognoses varied from one another and evaluated the model’s precision.

### 2.7 Independence and validity assessment of the prognostic model

To calculate the probabilities of OS at 1, 3, and 5 years, we developed a nomogram combining the risk score, age, gender, pathological stage, and other clinical parameters as independent prognostic factors. In the meantime, survival curves were plotted using the Kaplan-Meier method for prognostic reasons, and log-rank tests were run to assess the statistical significance. The receiver operating characteristic (ROC) curves, calibration curves, and concordance index curves were also used to assess the nomogram’s prediction accuracy.

### 2.8 Tumor immunity and immunotherapy

We next determined the degree of immune infiltration for BC patients in the TCGA database from the TIMER 2.0 database, which contains the results of 7 evaluation methods. These data were applied to quantify the relative fractions of immune cell infiltration in the TME in the form of heatmaps. We were able to deduce tumor purity and the presence of stromal and immune cells in malignant tumor tissues from the expression profiles. The “estimate” R package allows users to determine the relative abundance of stromal cells, immune cells, and tumor cells (28) and then compare these values across different risk categories. A higher score indicates a larger proportion of components in the TME. Additionally, immune checkpoints are comprised of various molecules that are expressed on immune cells and can regulate the level of immune activation. They play a crucial function in preventing excessive immunological activation. We compared the levels of expression in both groups of well-known immune checkpoint genes (ICGs) that were extracted from the literature. Correlations between ICGs expression and model genes and risk scores were further explored. The Cancer Immunome Atlas (TCIA) database was used to retrieve the Immunophenoscores (IPS) for BC. The online Tumor Immune Dysfunction and Exclusion (TIDE) algorithm was used to assess the potential responsiveness to ICI treatment (http://tide.dfci.harvard.edu/) (29, 30).

### 2.9 Tissue sample collection and cell lines culture

The tissue samples collected from the First Affiliated Hospital of Nanjing Medical University were approved by the Medical Ethics Committee of the hospital (2010-SR-091) and were kept at -80°C. The clinical sample information of 20 pairs of patient tissues were presented in **Supplementary Table S1**. All samples were taken with the patient’s consent. A total of ten pairs of samples were collected from BC patients undergoing tumor resection between February 2021 and March 2021 (tumor tissue (T) and precancerous tissue (N)). Human BC cell lines (MDA-MB-231, MCF-7) were purchased from the Cell Resource Center of Shanghai Life Sciences Institute, and these cells were cultured in DMEM (Gibco BRL, USA). Cells were cultured in a 10% fetal bovine serum (Gibco BRL, USA), 100U/mL penicillin, and 100μg/mL streptomycin in 95% humidity and 5% CO_2_ at 37°C.

### 2.10 RT-qPCR

Total RNA was extracted from tissues or cell lines using TRIzol as directed by the manufacturer (15596018, Thermo). cDNA was then synthesized using the PrimeScript™RT kit (R232-01, Vazyme). The Real-time polymerase chain reaction (RT-PCR) was performed by SYBR Green Master Mix (Q111-02, Vazyme), and the expression levels were counted with the 2^−ΔΔCt^ method. The expression of each mRNA was standardized by the expression level of mRNA GAPDH. All primers were supplied by Tsingke Biotech (Beijing, China), and detailed primer sequences were in **Supplementary Table 2**.

### 2.11 RNA interference

A small interfering RNA (siRNA) probe against SNX3 was developed and synthesized by Ribobio (Guangzhou, China). All transfections were carried out with Lipofectamine 3000 (Invitrogen, USA). The siRNA sequences for SNX3 are provided in **Supplementary Table 2**.

### 2.12 EdU

5-Ethynyl-2’-deoxyuridine (EdU) assay was then performed under the manufacturer’s instruction (Ribobio, China). After incubating in a cell incubator for 2 hours, we rinsed the cells with PBS and then immersed them in 4% paraformaldehyde at room temperature for 10 min with 0.5% Triton-X-100. Apollo^®^ fluorescent dye was used for staining. The number of proliferating cells was analyzed under an inverted microscope.

### 2.13 Healing assay

Transfected cells were seeded into 6-well plates and incubated in a cell incubator until 95% confluent. After serum starvation, one straight line was scraped with a sterile 20 μl plastic pipette tip and gently washed away unattached cells and debris twice with PBS in each cultured well. Eventually, we took photographs of the scratch wounds after 0h and 48h, and the ImageJ software measured the width of the scratches.

### 2.14 Colony formation

In a 6-well plate, we transfected 2×10^3^ cells per well. All cells were maintained for 2 weeks until the formation of visible colonies. The cells were rinsed twice with PBS and fixed for 15 minutes in 4% paraformaldehyde before Crystal violet (Solarbio, China) staining. The colonies were counted per well.

### 2.15 Transwell assay

Transwell experiments included cell migration and invasion experiments. In the upper chamber, 2×10^4^ cells per well were incubated in a serum-free medium. The lower chamber maintains 600μl of complete medium. The upper portion of the plate was either pre-coated or uncoated with Matrigel solution (BD Biosciences, USA) to evaluate the invasive and migratory capabilities of the cells. Cells were fixed with 4% PFA, stained with 0.1% crystal violet (Solarbio, China), and counted under a light microscope.

### 2.16 Statistical analysis

Software called GraphPad Prism (version 8.0) was used to analyze experimental data. Three independent experiments recorded the data as mean ± standard deviation (SD). We tested the comparisons among the groups with Student’s t-tests (*P<0.05, **P<0.01, ***P<0.001).

## 3 Results

### 3.1 Single-cell sequencing data of BC analysis

**Figure 1:**
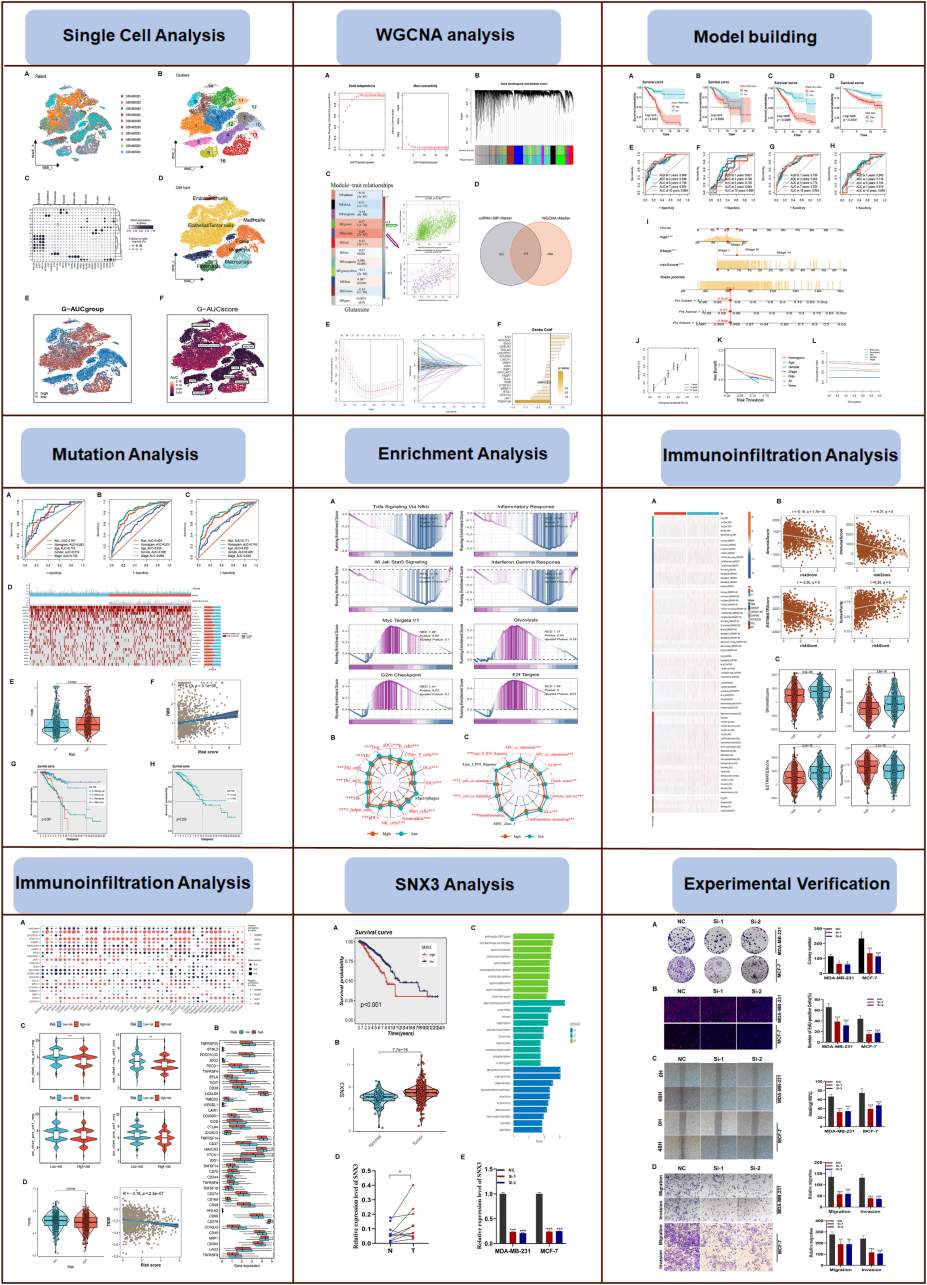
The flowchart of this study.

**Figure 2:**
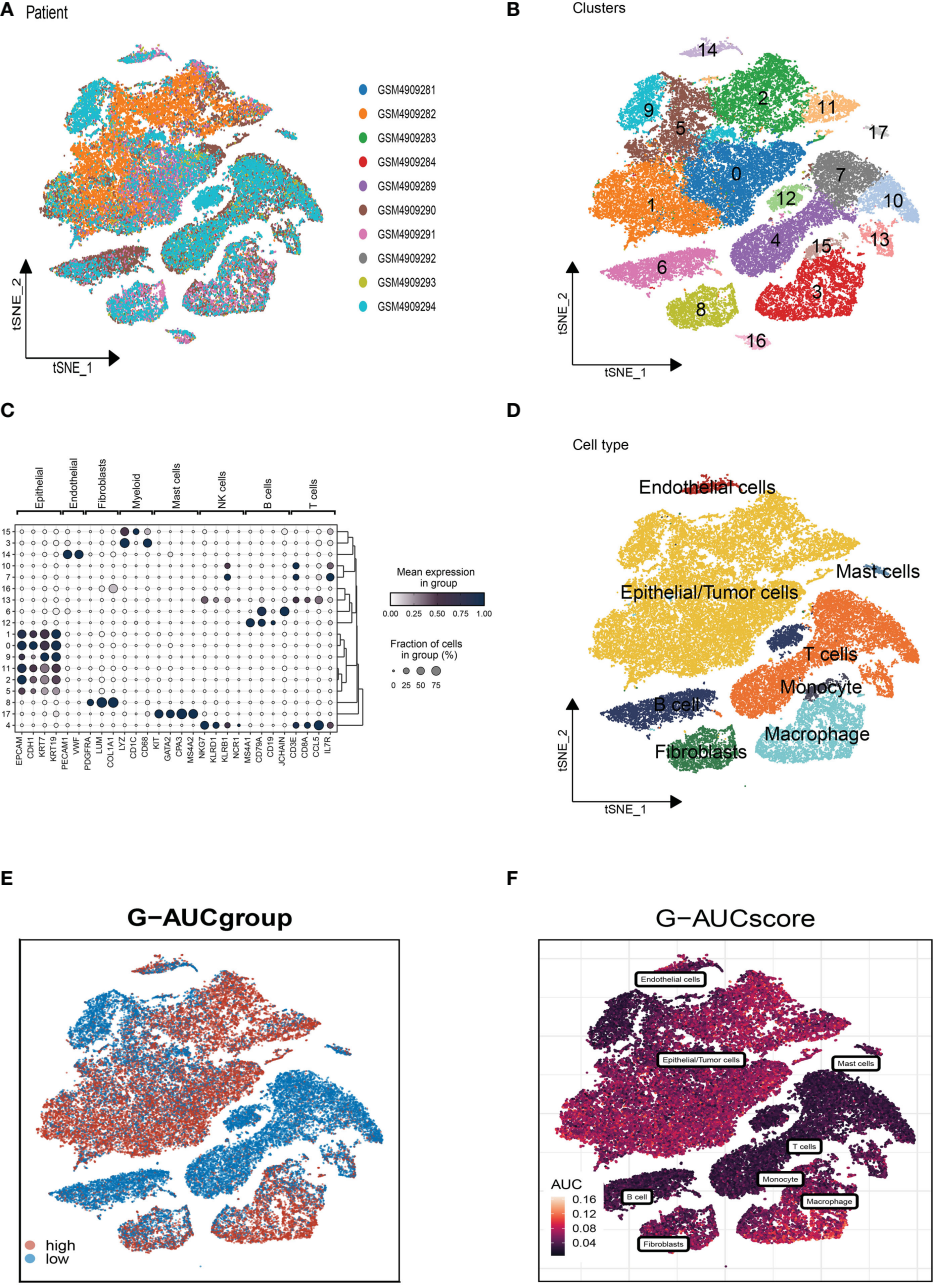
Annotation of cell subsets from single-cell sequencing data and identification of differentially expressed genes. **(A)** The cell distribution of the samples showed no significant batch effect. **(B)** The dimension reduction cluster analysis results are shown in the tSNE diagram. **(C)** The expression of cell type marker genes. **(D)** The tSNE map indicates that BC samples can be annotated as 8 cell types in the TME (different colors represent different cell types). **(E, F)** All cells were scored according to glutamine-associated genes (GRG) and were divided into high and low groups.

The flow chart of this study was shown in **Figure 1**. On the single-cell data set, we conducted quality control. To confirm the validity of the cell samples, as seen in **Supplementary Figure S1A**, we removed some cells and restricted the percentage of mitochondrial genes, ribosomal genes, and red blood cell genes. Sequencing depth and total intracellular sequences exhibit significantly substantial positive associations (R=0.92, **Supplementary Figure S1B**). **Supplementary Figure S1C** shows that TCGA and GEO cohorts independently, with significant batch effect. After removing the batch effect, better results were obtained (**Supplementary Figure S1D**). The study contained 10 samples, and each sample’s cell distribution was largely constant. This suggests that there was no noticeable batch impact on the samples, which might be used for further analysis (**Figure 2A**). Subsequently, all cells were classified by the dimensionality reduction algorithms, namely, t-SNE into 18 clusters (**Figure 2B**). The expression of cell-type marker genes is shown in **Figure 2C**. **Figure 2D** illustrated the distribution of each cell population with a t-SNE plot. A total of eight cell types can be found, such as Endothelial cells, Mast cells, Fibroblasts, and Tumor cells. Using the “AUCell” R package, the GRGs activity of each cell line was discovered to explore the GRGs expression characteristics (**Figure 2E**). Higher AUC values were seen in cells that expressed more genes, and these cells were primarily orange-colored Macrophage cells (**Figure 2F**). All cells were assigned an AUC score for the corresponding GRGs and divided into two groups (high-and low-Glutamine-AUC groups) by AUC score median values.

### 3.2 Weighted co-expression network analysis and construction

**Figure 3:**
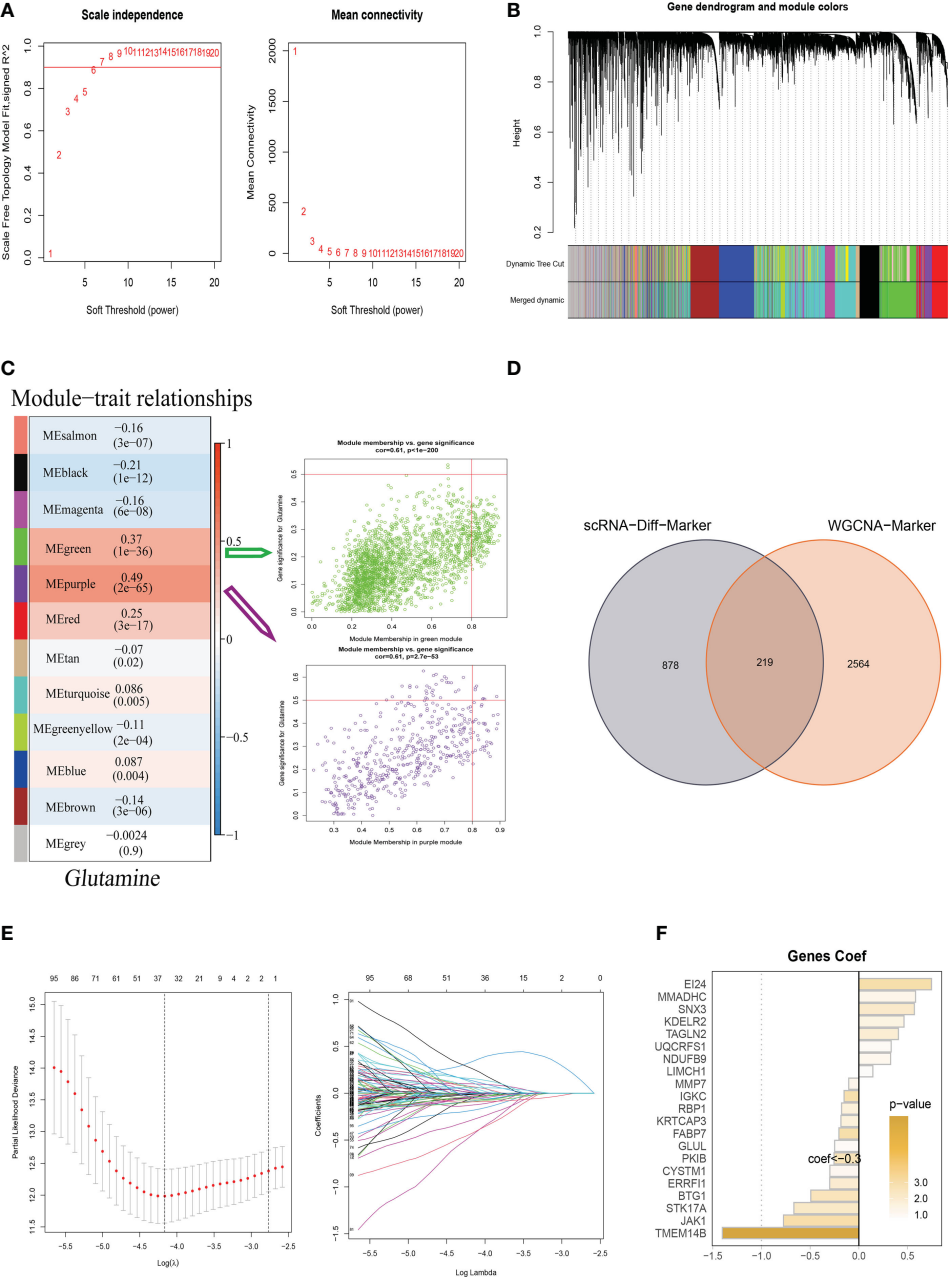
Weighted Co-Expression Network Analysis and construction Glutamine-Related Prognostic Model. **(A–C)** Weighted Co-Expression Network Analysis. The green and purple modules were most associated with glutamine, of which 2,783 genes were extracted. **(D)** The intersection of genes obtained in single-cell analysis and bulk-RNA analysis. **(E)** LASSO Cox regression analysis to develop the prognostic model. **(F)** The role of twenty-one model genes.


WGCNA was used to look for gene sets that were covarying with glutamine in more detail. As seen in **Figure 3A**, the data is more consistent with the power-law distribution and the mean connectivity tends to be stable when the soft domain value is 6; this makes the data suitable for further study. As seen in **Figure 3B**, 12 non-gray modules were generated after merging the modules with a similarity lower than 0.25 and setting the minimum number of modules to 100 and deepSplit to 2. According to **Figure 3C**, a total of 12 non-gray modules were obtained. We discovered that the green and purple modules, which each contained 2,783 genes, were most closely related to GM (COR = 0.61, *P <*0.001). To further explore how GRGs relate to the prognosis of BC patients, we intersected the most relevant genes affecting glutamine metabolic activity obtained in single-cell and Bulk-RNA analysis and finally, 219 genes were used for subsequent analysis (**Figure 3D**). We used the training set in TCGA-BC for model construction, and prognostic genes were obtained by univariate analysis (*P*<0.01). Next, LASSO Cox regression and multivariate regression analysis were employed to develop the prognostic model (**Figure 3E**). A total of twenty-one model genes (EI24, MMADHC, SNX3, KDELR2, UQCRFS1, NDUFB9, LIMCH1, MMP7, IGKC, RBP1, KPTCAP3, FABP7, GLUL, PKIB, CYSTM1, ERRFI1, BTG1, STK17A, JAK1, TMEM14B) were finally screened out under optimal regularization parameters. The prognostic model was calculated as follows:

$$risk\;score=\sum_{n=i}^{k}\left(Coef_{i}\exp i\right)$$

Coefi and Expi represented the coefficient and expression of each model gene, respectively, and the risk score for each sample was calculated by the above formula. By using the aforementioned formula, the risk score for each sample was determined. Based on median values, patients were split into high-risk and low-risk categories. Of the twenty-one genes used to construct the model, eight were risk factors and thirteen were protective factors (**Figure 3F**).

### 3.3 Validation of glutamine-related prognostic model and construction of a nomogram

**Figure 4:**
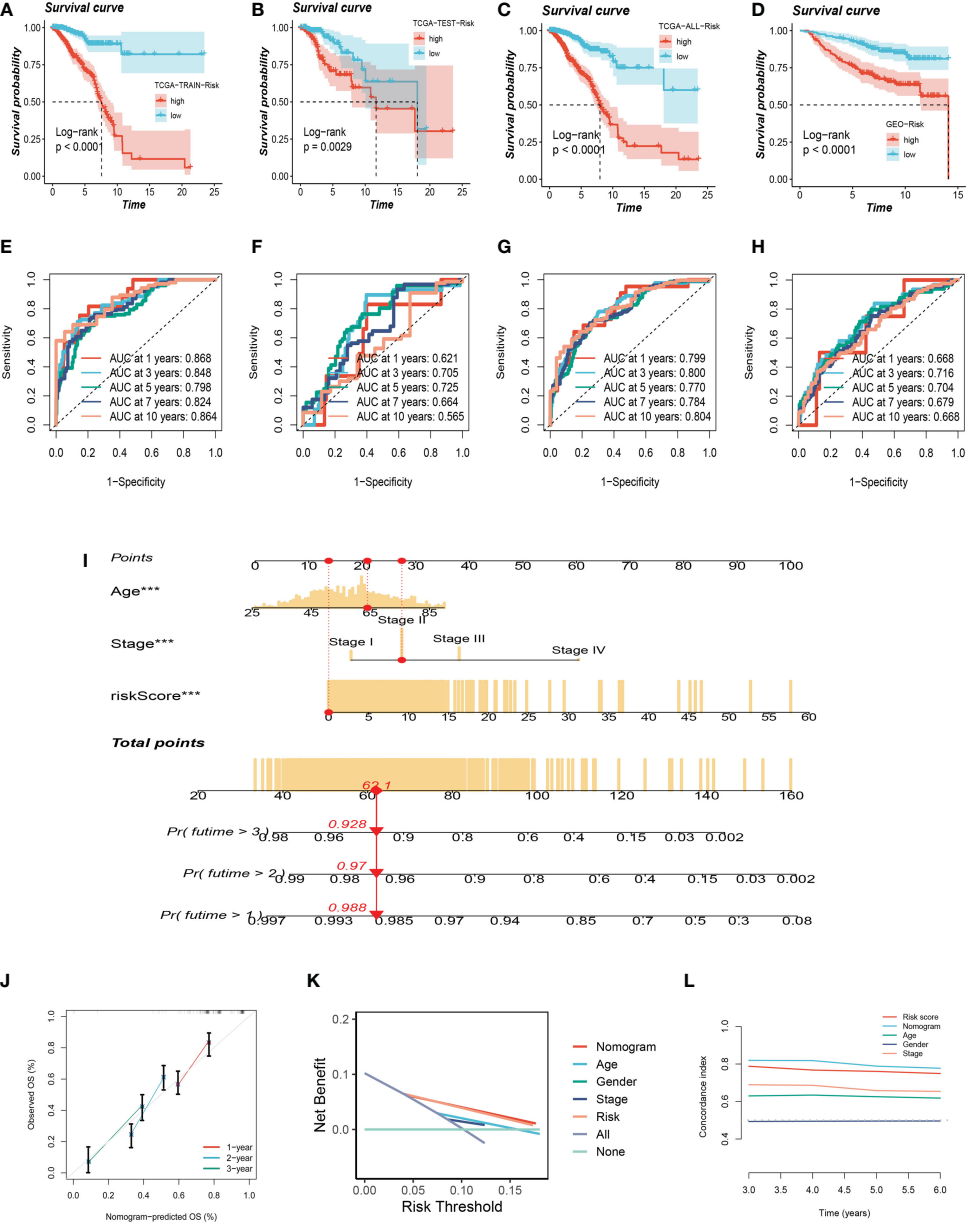
Validation of Glutamine-Related Prognostic Model. **(A–C)** Survival analysis in the TCGA train, test, and entire cohort (P <0.001). **(D)** Survival analysis in the GEO test cohort. **(E–G)**The area under the curve (AUC) values for the TCGA train, test, and full cohort. **(H)** the areas under the curve at 1, 3, and 5 years for the GEO test group. **(I)** Nomogram to assess the risk of BC patients. **(J)** Calibration curves for the nomogram. **(K)** Decision curve. **(L)** Concordance index study. The *** represents P<0.001.

To testify to the credibility of the glutamine-related prognostic model, we performed a survival analysis. For patients in the training, testing, and all cohort, the overall survival rate of high-risk group patients decreased more dramatically compared with the low-risk group (**Figures 4A–C**). We also obtained the same result in the external validation GEO cohort (**Figure 4D**). We performed ROC curve analysis in both the training cohort and the test cohort to further investigate the precision of glutamine in the assessment of the prognosis of BC patients. The areas under the 1, 3, and 5-year ROC curve (AUC) were: training cohort 0.868, 0.848, and 0.798, testing cohort 0.612, 0.705, 0.725, and all cohort 0.799, 0.800, 0.770 respectively (**Figures 4E–G**). The AUC of the external validation GEO cohort was 0.668, 0.716, and 0.704 in 1, 3, and 5 years, which further confirmed our PRS’s predictive ability (**Figure 4H**).

**Figure 5:**
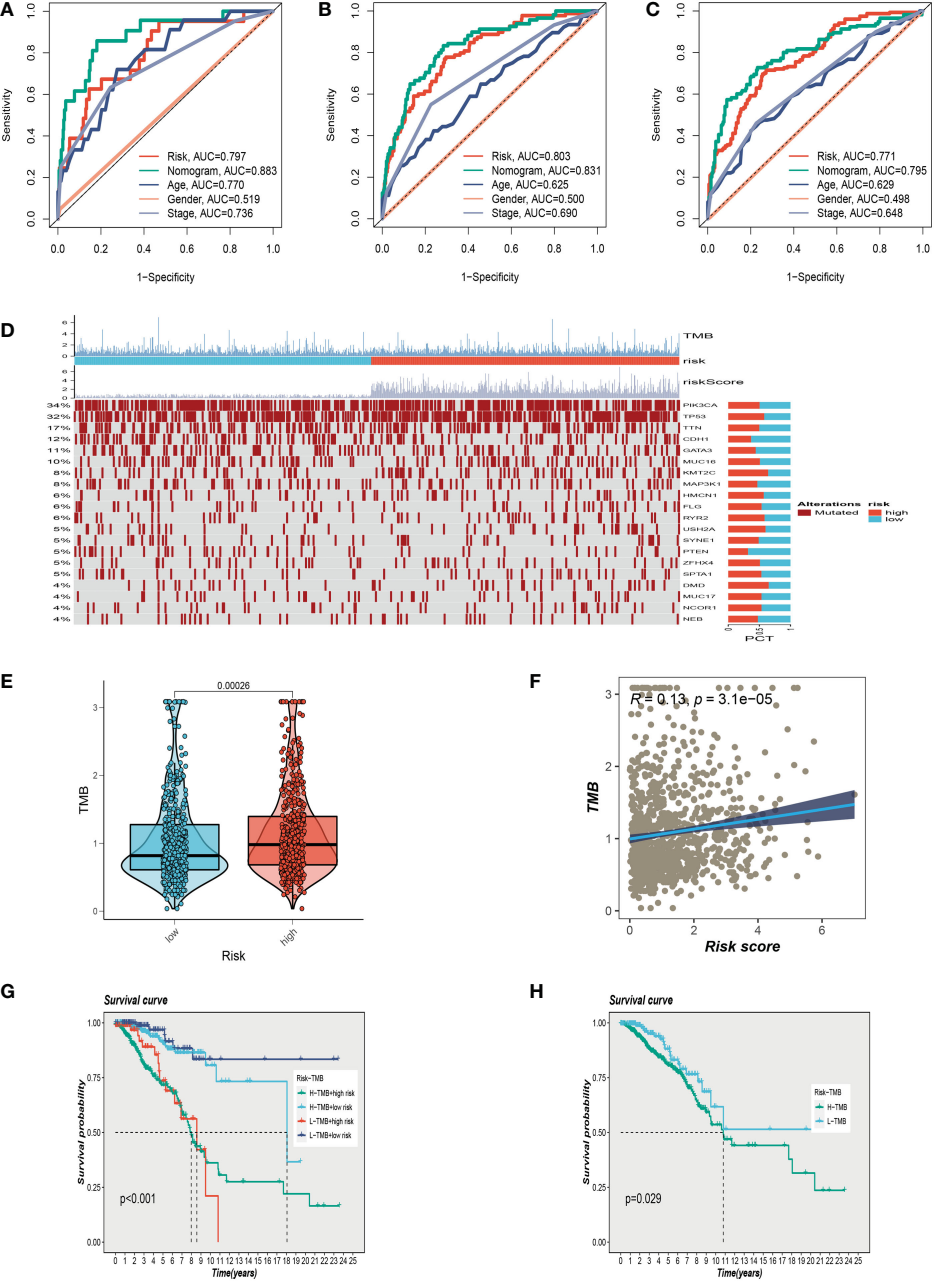
Clinical correlation analysis and gene mutation analysis. **(A–C)**Prognostic ROC analysis in 1, 3, and 5 years, respectively. **(D)** The representative gene variants in the groups at high and low-risk groups. **(E)** The two risk groups have differences in tumor mutation burden (TMB) levels. **(F)** The correlation between TMB and risk score. **(G, H)** Correlation analysis between TMB and prognosis.

Using clinical information and a risk score, a nomogram was created to more accurately quantify the risk of BC patients (**Figure 4I**). The nomogram can help determine patient risk more accurately and direct future treatment decisions. The calibration plot is used to testify that the nomogram is consistent with our prediction, which showed good agreement with the actual outcome (**Figure 4J**). We also carried out the decision curve and concordance index study, which determines the area of each clinical feature and None’s horizontal axis to assess the clinical decision value. Results indicated that this nomogram’s efficacy was superior to that of other clinical indicators, indicating that it is effective in forecasting patients’ prognoses and can serve as a clinical decision-making tool (**Figure 4K, L**). Prognostic ROC analysis was carried out to thoroughly assess the accuracy of this nomogram. According to the findings, the area under the curve (AUC) was 0.797, 0.803, and 0.771 in 1, 2, and 3 years, respectively (**Figures 5A–C**).

### 3.4 Mutation landscape analysis

We examined representative gene variants in the groups at high- and low risk (**Figure 5D**). Genes such as TP53, KMT2C, HMCN1, USH2A, and DMD had the top five mutation frequencies in the high-risk group. The top five genes with the highest mutation frequencies in the low-risk group were PIK3CA, CDH1, MAP3K1, PTEN, and GATA3 respectively. Tumor mutation load (TMB) was significantly different between the two groups, and the mutation load in the high-risk group was higher than that in the low-risk group (**Figure 5E**). Further analysis showed that with the increase of risk score, tumor mutation load also increased correspondingly, and the two showed a positive correlation (**Figure 5F**). High TMB is closely associated with poor survival outcomes. After dividing patients into subgroups, the high-risk/high-TMB group showed a poorer survival outcome (**Figures 5G, H**).

### 3.5 Biological function and pathway analyses

**Figure 6:**
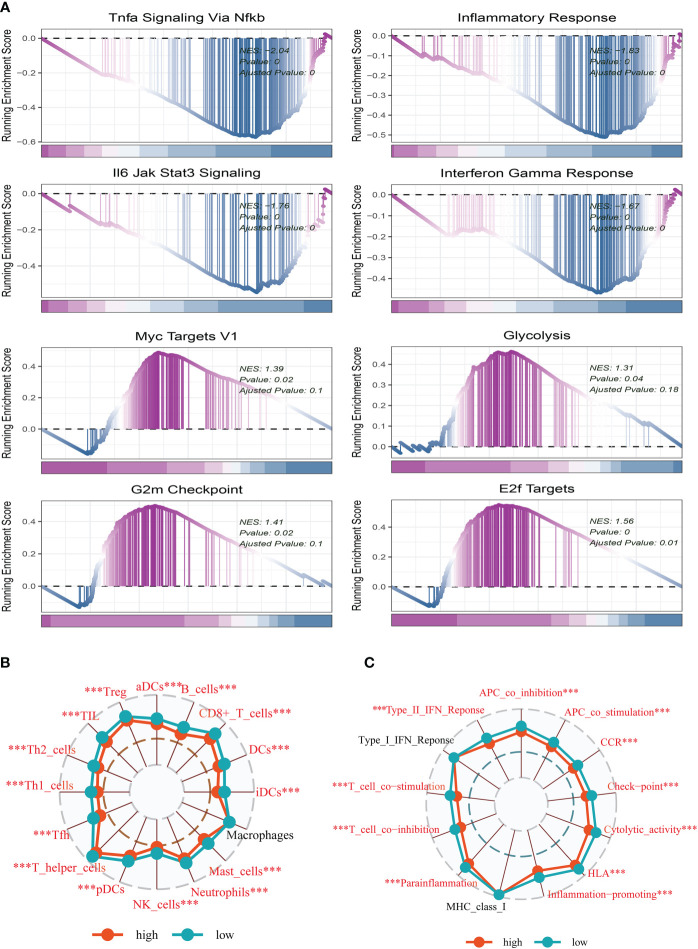
Enrichment analysis and functional annotation. **(A)** GSVA shows the enrichment of hallmark gene sets in different risk subgroups. **(B, C)** The ssGSEA algorithm was used to evaluate the differences in immune cells and immune-related functions between high- and low-risk subgroups. The *** represents P<0.001.

To explore the underlying mechanism that could lead BC patients in the high-risk group to a poor prognosis. Analysis of hallmark pathway gene signatures highlighted that the high-and low-risk groups showed some differences. A direct comparison of Risk-High versus Risk-Low revealed the enriched signatures in the high-risk group included Glycolysis, Myc Targets V1, G2M checkpoint, and E2F targets. Characteristics of enrichment in the low-risk group included Tnfa signaling *Via* NF-κB, inflammatory response, IL6 jak stat3 signaling, and interferon-gamma response (**Figure 6A**). Glycolysis is an essential condition for the occurrence and development of tumors (7, 31). High-risk samples may present a worse prognosis for BC patients by upregulating the glycolytic pathway. High MYC targets v1 and v2 scores were related to both increased pro- and anti-cancerous immune cell infiltration in ER-positive BC (32). Extremely crucial nuclear transcription factors involved in controlling the cell cycle are encoded by the E2F family (33, 34). Triple-negative breast cancer tumorigenicity is aided by transcriptional regulation of CCNA2 expression by E2F1 (35). To control cell proliferation, the G2M checkpoint also functions as a cell cycle regulatory route. As a result, these pathways, which were more prevalent in the high-risk group, may play a crucial role in controlling tumor development in BC. To explore the TME of high-and low-risk group samples, we used ssGSEA to evaluate the composition of immune cells between two risk groups. **Figures 6B, C** show that in the tumor microenvironment of patients in the high-risk group, immune cell infiltration is generally lower than that in the low-risk group.

### 3.6 Immune landscape and immunotherapy

**Figure 7:**
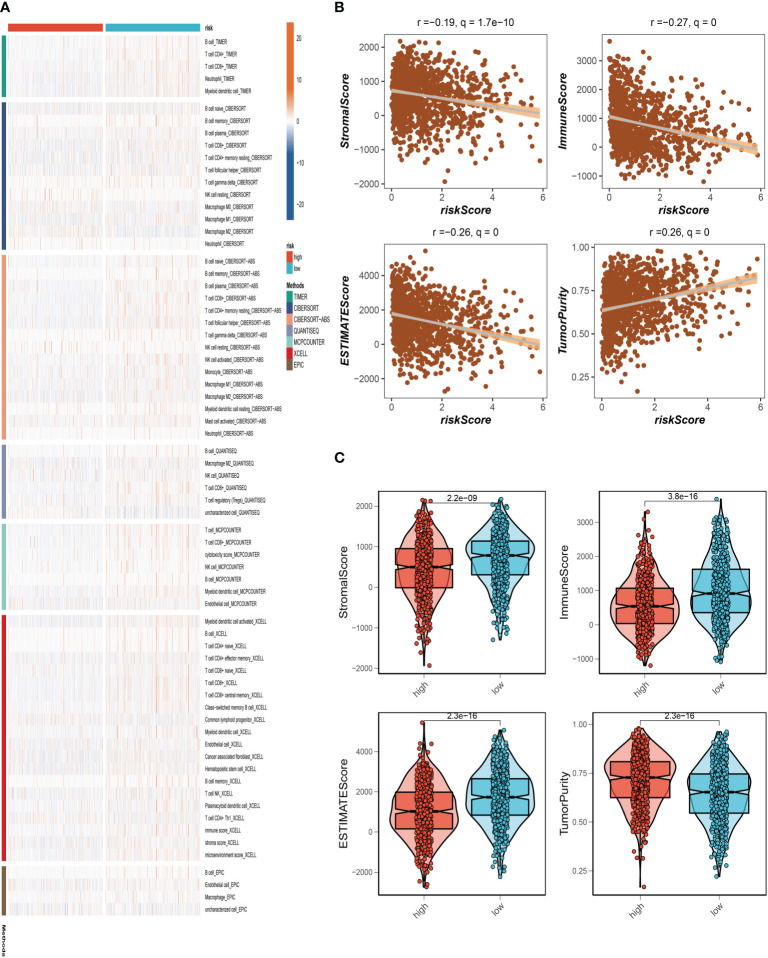
Analysis of immune microenvironment. **(A)** The distribution and association of the 22 tumor-infiltrating immune cells (TICs) in the TCGA-BC cohort. **(B, C)** Correlation analysis of immune score and risk score, ESTIMATE score and risk score, Stromal score and risk score, tumor purity and risk score.

To further understand the distribution and correlation of the relative content of 22 tumor-infiltrating immune cells (TICs) in the TCGA-BC cohort, we measured the level of immune cell infiltration in each sample using the CIBERSORT method. We found that immune cell infiltration was overall higher in the low-risk group than in the high-risk group. NK cells and T cell CD4+ infiltrated more in the high-risk group. (**Figure 7A**). The low-risk group then had higher stromal scores, immunological scores, and ESTIMATE scores (*P*<0.001), indicating a higher overall immune level and immunogenicity of the TME in that group. We also looked at tumor purity, and the results showed a positive correlation between the two (**Figures 7B, C**).

### 3.7 Immune checkpoint analysis and immunotherapy response assessment

**Figure 8:**
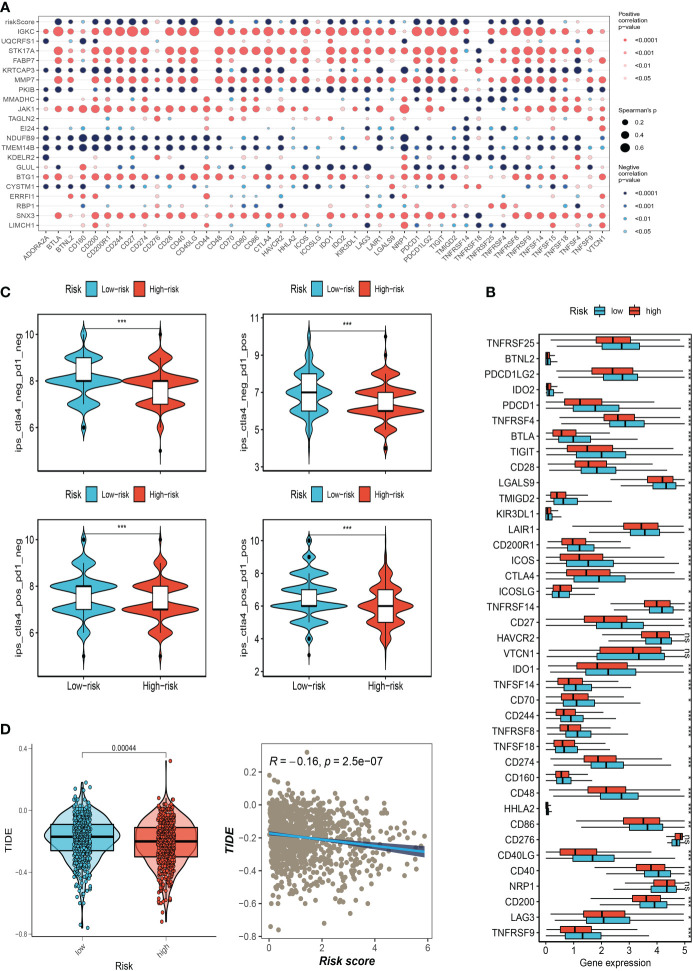
Correlation analysis of immune-checkpoint and treatment response. **(A)** Correlation between model gene and immune checkpoint. **(B)** Differences in the abundance of immune-checkpoint-related genes between high and low-risk groups. **(C)** Differences in IPS reactivity between high and low-risk groups. **(D)**The difference in TIDE scores between high and low-risk groups. (*P<0.05, **P<0.01, ***P<0.001). The ns indicates No significance.

We also examined the differences in immune checkpoint expression between the two groups because immunological checkpoints are crucial for the efficacy of immunotherapy in malignancies. The bubble map revealed the correlation between the model genes and 46 immune checkpoint genes (**Figure 8A**). IGKC, STK17A, FABP7, MMP7, JAK1, BTG1, and SNX3 were significantly correlated with immune checkpoint genes. 37 immune checkpoint genes were significantly upregulated in low-risk people. The expression of only one immune checkpoint gene ICOSLG was observed in the high-risk group and was called high in the low-risk group (**Figure 8B**). Patients with this subtype of tumor might benefit from targeted therapy against immunological checkpoints that have increased expression. Furthermore, IPS can contribute to screening patients who are susceptible to immunotherapy. In our research, the low-risk subtype has higher IPS and blocker scores than the high-risk subtype, highlighting that low-risk patients may be more susceptible to immune checkpoint inhibitors (ICIs) treatment and derive more significant benefits (**Figure 8C**). Regarding how TMB and immunotherapy interact, to determine if patients with various risk patterns respond to immunotherapy differently, a tumor immune dysfunction and exclusion (TIDE) analysis was performed. According to the findings, the high-risk group responded to immunotherapy better since they had a lower TIDE score and risk score was negatively correlated with TIDE (**Figure 8D**).

### 3.8 Expression of SNX3 in BC samples

**Figure 9:**
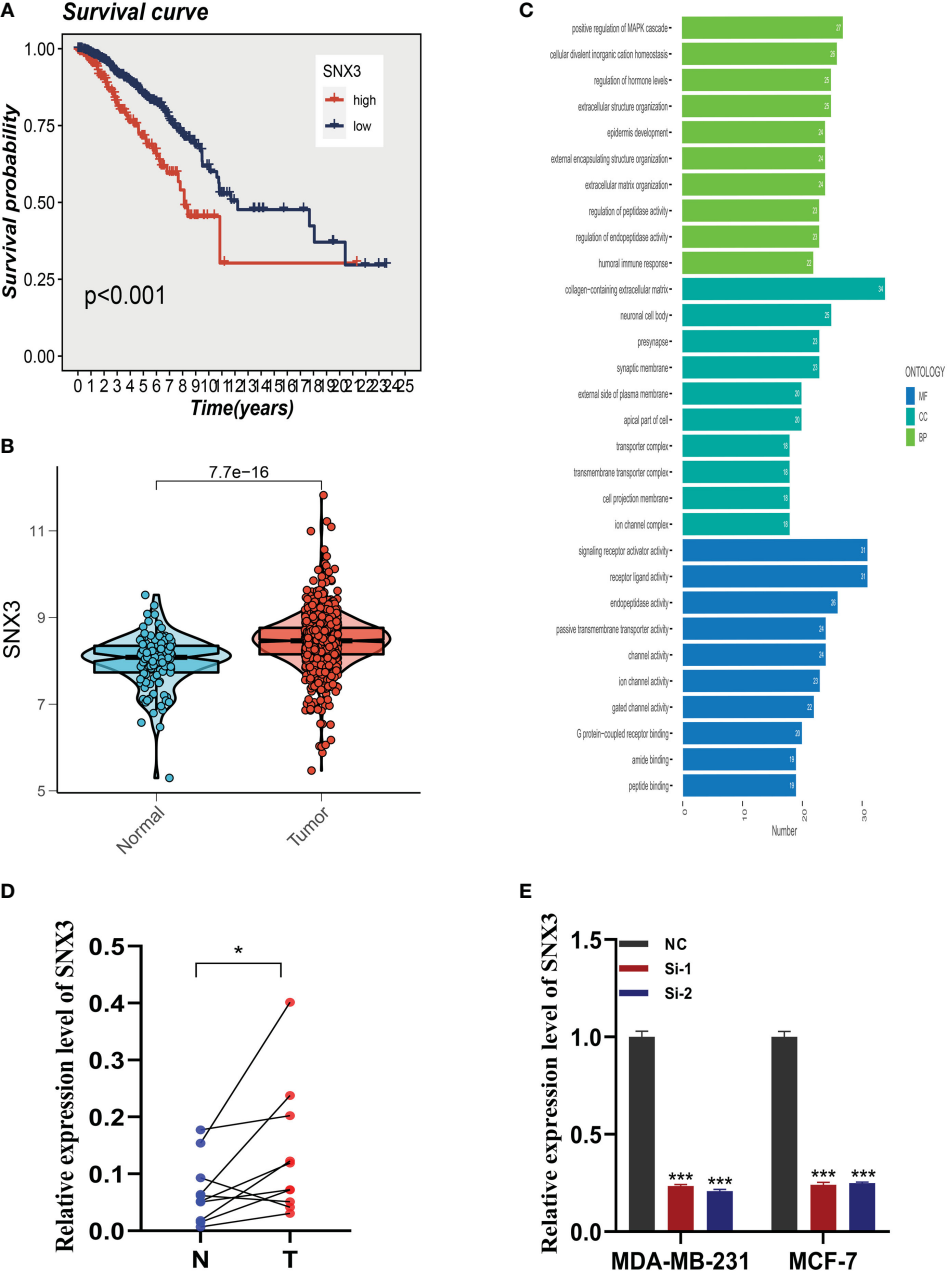
Expression analysis and experimental validation of SNX3. **(A)** Expression of SNX3 in normal and tumor tissues of BC. **(B)** The overall survival (OS) analysis of SNX3 in the TCGA cohort. **(C)** GO enrichment analysis of SNX3. **(D)** PCR assay of clinical samples. SNX3 was highly expressed in BC. **(E)** SNX3 was knocked down in MCF-7 and MDA-MB-231. (*P<0.05, **P<0.01, ***P<0.001).

Analysis of the survival prognosis of SNX3 in the TCGA showed that BC patients with high expression of SNX3 had a poor prognosis (**Figure 9A**). At the same time, we found that compared with normal tissues, SNX3 has a higher expression level in BC tissues (**Figure 9B**). As is shown in the bar plot of the GO enrichment analysis of SNX3 (**Figure 9C**). We did the same validation with ten pairs of BC tissue samples from our hospital. In clinical samples, we observed similar expression trends (**Figure 9D**). **Figure 9E** indicated that the expression of SNX3 was significantly decreased in transfected MDA-MB-231 and MCF-7 cells.

### 3.9 Experimental validation of SNX3

**Figure 10:**
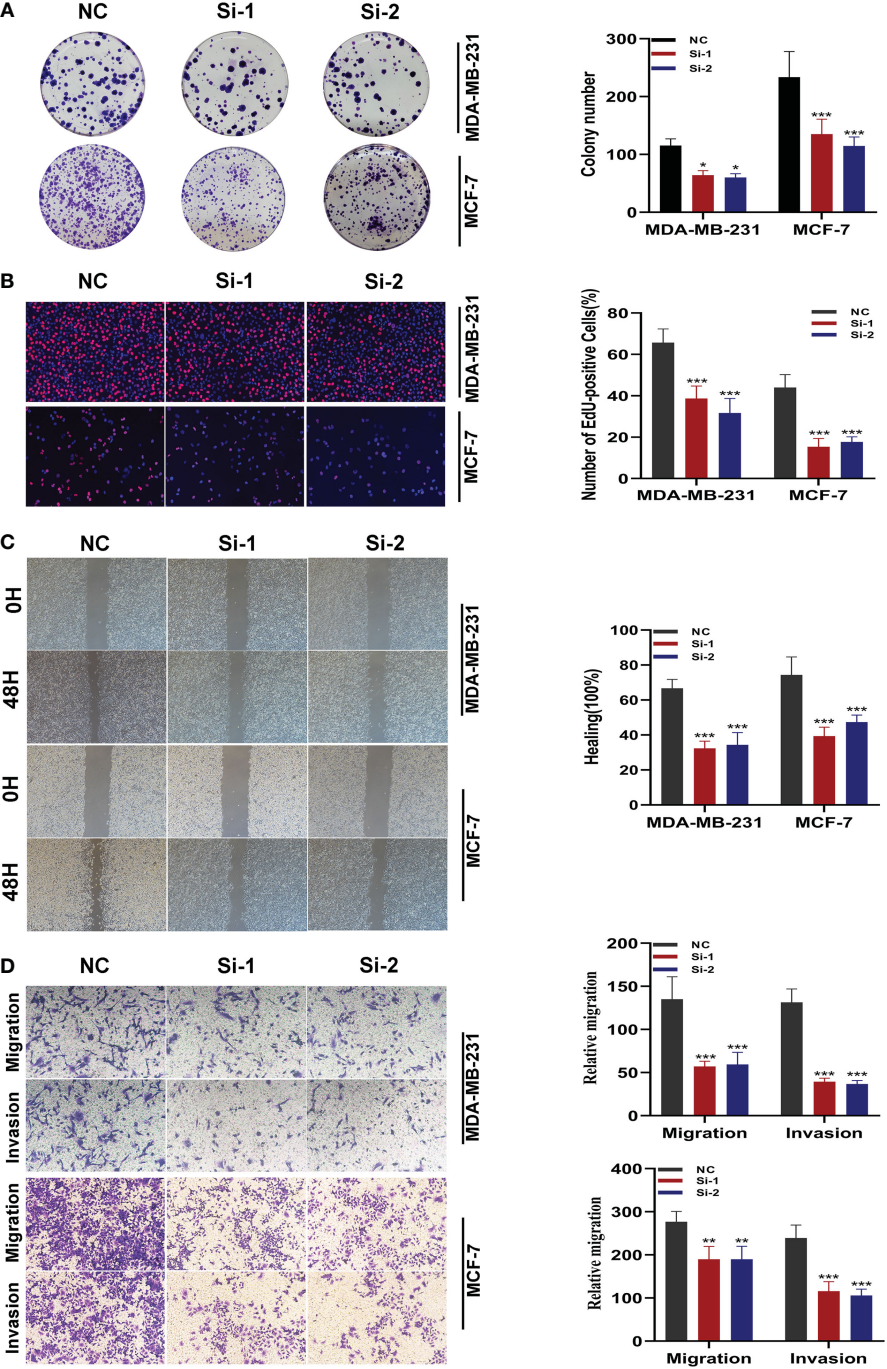
*In vitro* experiment after SNX3 knockdown. **(A)** After SNX3 knockdown, the cloning ability of MDA-MB-231 and MCF-7 cell lines decreased significantly. **(B)** EdU test. After SNX3 knockdown, the proliferation ability of MDA-MB-231 and MCF-7 cell lines decreased significantly. **(C)** Healing test. After SNX3 knockdown, the migration ability of MDA-MB-231 and MCF-7 cell lines decreased significantly. **(D)** Transwell assay. After SNX3 knockdown, the migration and invasion abilities of MDA-MB-231 and MCF-7 cell lines were significantly decreased. (*P < 0.05, **P < 0.01, ***P < 0.001).

After the knockdown of SNX3, MDA-MB-231 and MCF-7 cell lines significantly reduced their ability to form colonies (**Figure 10A**). In the 5-ethynyl-2 deoxyuridine (EdU) assay, after the knockdown of SNX3, the proliferation of MDA-MB-231 and MCF-7 cell lines were greatly reduced, suggesting that the SNX3 may progress proliferation (**Figure 10B**). Healing and transwell assay in **Figures 10C, D** showed that after SNX3 knockdown, cells migrate and invade more slowly than disordered siRNA, indicating that SNX3 knockdown may weaken the migration and invasion of MDA-MB-231 and MCF-7 cell lines. The difference was statistically significant.

## 4 Discussion

Breast cancer has become cancer with the highest incidence in the world, and its heterogeneity makes the classification and treatment of BC enter the era of precision treatment (1). With the success of immunotherapy, BC, which was previously considered “weakly immunogenic”, has also entered the stage of immunotherapy. Immunotherapy of BC has proved to be a challenge in the era of personalized treatment. The interaction between cancer cells and the immune system is a complex, dynamic, and constantly changing process (36). Unlike targeted therapy and endocrine therapy, which effectively guide attacks by identifying targets with biological markers, there are no therapeutic markers for immunotherapy. Up to now, predictors of BC immunotherapy response have included PD-L1 status, TMB, immunogenomic features, and TILs; however, none of them has sufficient evidence to be used as a stratification factor (37). Therefore, further exploration of biological mechanisms and prognostic biomarkers for BC may provide an opportunity to identify BC subtypes and thus improve precision-focused treatment of BC in the future.

The metabolism of amino acids plays a significant role in controlling the immune response in the tumor microenvironment (38). Unlike conventional cancer treatment modalities, immunotherapy reverses the immune balance in the tumor microenvironment by restoring the proliferation and effector functions of immune cells and ultimately assists the autoimmune system in killing tumor cells (39). Clinical studies have demonstrated that the complexity of etiology, individual variances, and the variety of tumors are all strongly correlated with the success of immunotherapy. Therefore, it is important to further investigate the role of metabolic reprogramming in TME formation and maintenance to improve tumor immunotherapy. Metabolic phenotypes evolve with cancer and new dependencies emerge in the context of treatment resistance and metastasis, and drugs that target the reprogramming of amino acid metabolism within the tumor microenvironment in concert with cancer immunotherapy have far-reaching implications in clinical treatment (40). Initially, the goal of tumor immunotherapy was to increase the signaling pathways that T cells activate. Immune checkpoint blockade therapy also improves tumor infiltration and T cell effector functions by reprogramming amino acid metabolism in addition to tumor immunotherapy’s targeting of glucose and lipid metabolism (41). For example, increased uptake of glutamine during T-cell activation and PD-1 signaling resulted in reduced expression of the corresponding transporter proteins SLC38A1 and SLC38A2 by T cells and concomitantly reduced catabolism of branched-chain amino acids (including valine and leucine) (42). Therefore, inhibiting the immune checkpoint receptor releases the restriction on T cell differentiation by reprogramming glutamine metabolism, and tissues from patients who received immune checkpoint blocker showed increased T cell infiltration as well as upregulation of interferon regulatory gene expression (ICB) (43). Interferon IFN-y can down-regulate the expression of transporter proteins SLC7A11 and SLC3A2 in tumor cells, inhibit the input of cysteine required for glutathione synthesis, cause intracellular glutathione depletion, and thus indirectly lead to glutathione peroxidase-4 (GPX4) inactivation and ultimately induce iron death in tumor cells (44). Thus, the close link between amino acid metabolism and T-cell immunity has led to the progressive emergence of amino acid metabolic reprogramming as an important target for cancer immunotherapy.

We constructed a novel survival risk signature by Glutamine metabolism-related genes, which performed well in both TCGA internal and GEO external validation cohorts. The AUC values exceeded 0.8 at 1, 3, and 10 years, while a maximum AUC value of 0.868 was detected at 1 year. In addition, a nomogram combining prognostic models and clinicopathological factors was established. Compared with other traditional features such as TNM, the PRS showed the best accuracy and discriminative power in predicting OS.

T cells and macrophages are the main representatives of the lymphoid and myeloid lineages of the immune system, respectively. While glutamine stimulates the polarization of M2 macrophages *via* the Gln-UDP-GlcNAc pathway and a-ketoglutarate generated by glutamine degradation, amino acid metabolism can also drive the activation and proliferation of T cells (45). The data confirm that M2 macrophages have tumor-promoting effects *in vitro*, and our study found more M1 macrophage infiltration in the bottom-risk group and more M2 macrophages in the high-risk group, suggesting a rationale for developing cancer therapies that target TAMs (46). Tumor cells must upregulate extracellular absorption in addition to glutathione synthesis to preserve tumor cell viability because they can regulate ROS levels through glutathione and NADPH created by glutamine metabolism to prevent chromosomal instability brought on by high levels of ROS (11). Our results suggest that low PRS patients respond better to immunotherapy. Therefore, glutamine metabolism and immunotherapy may have an exceedingly close relationship. Studies have shown that patients with high TMB have significantly higher rates of both progression-free survival and overall survival. Regardless of tumor type and detection modality, TMB is a reliable biomarker for predicting the effect of immunotherapy (47). In our study, we found that TMB levels were positively correlated with risk scores, suggesting that patients in the high-risk group may be more suitable for immunotherapy. TIDE stands for tumor immune dysfunction and rejection. It is a computational framework for assessing the likelihood of tumor immune escape in the gene expression profile of tumor samples (48). A higher TIDE score implies a higher likelihood of immunosurveillance escape and a lower success rate of immunotherapy. In our study, the TIDE score was found to be negatively correlated with the risk score, again suggesting that patients in the high-risk group may be more suitable for immunotherapy. Next, we evaluated the correlation between the genes used to construct the models and immune checkpoints. We found that IGKC, STK17A, FABP7, MMP7, JAK1, BTG1, and SNX3 have a strong correlation with immune checkpoints, and these model genes may become the targets of immunotherapy for BC patients.

Immune Checkpoint is a class of immunosuppressive molecules that are expressed on immune cells and can regulate the degree of immune activation. Tumor cells express substances that activate immune checkpoints, which, once activated, prevent antigen presentation to T cells, blocking antigen presentation in tumor immunity and inhibiting T cell immune function. Sorting linker protein 3 (SNX3) is a high-risk gene with a strong correlation with immune checkpoints in our construct signature. Therefore, we decided to perform *in vitro* experiments on this gene to explore its effect on BC. We found that SNX3 was highly expressed in BC tissues through TCGA database analysis; meanwhile, BC patients with high SNX3 expression had poorer survival. Our *in vitro* experiments showed that the knockdown of SNX3 expression significantly reduced the activity, invasion, and migration ability of BC cells. This adds to the evidence that SNX3 plays a role in BC. Many previous studies have shown that SNX3 has a function in malignant tumors. Through the miR-520a-3p/SNX3 axis, LINC01614 accelerates the progression of osteosarcoma (49). Through the β-linked protein pathway, SNX3 prevents the migration and invasion of colorectal cancer cells by reversing the epithelial-to-mesenchymal transition (50). In our study, SNX3 was also found to be a potential target for BC.

## 5 Conclusions

In conclusion, our results suggest that the model constructed with GRGs can well predict the prognosis of BC patients. In addition, we have validated the function of SNX3 in BC through cellular experiments and screened candidate Immune checkpoint inhibitors for BC. These findings may provide insights for the development of new treatment strategies for BC.

## Data availability statement

The original contributions presented in the study are included in the article/**Supplementary Material**. Further inquiries can be directed to the corresponding authors.

## Ethics statement

The studies involving human participants were reviewed and approved by Medical Ethics Committee of Nanjing Medical University. The patients/participants provided their written informed consent to participate in this study.

## Author contributions

HX and YX provided the idea for the project and participated in the design of the experimental scheme. SP and PZ performed data analysis and experimental operations. HC, SZ, and YD performed repeated experiments. LY, YK, and MZ contributed to data collection and analysis. HX and YX confirm the authenticity of all the raw data. All authors contributed to the manuscript and approved the submitted version.
