# Supplementary material for: Integrating single-cell RNA-seq and bulk RNA-seq to construct prognostic signatures to explore the role of glutamine metabolism in breast cancer
Source: Front Endocrinol (Lausanne). 2023 Feb 10;14:1135297. doi: 10.3389/fendo.2023.1135297 (PMC9950399; doi:10.3389/fendo.2023.1135297)
Supplement: Supplementary file 1 [file Table_1.docx]

| **Oligonucleotides** | **Nucleotide sequence (5'-3')** |
| --- | --- |
| **siRNA** |  |
| SiRNA-NC | GCUUCGCGCCGUAGUCUUA |
| Si-SNX3-1 | GAACGTTGTCTTCACATGTTT |
| Si-SNX3-2 | CAGTTTATAAACAAGGTCGCT |
| **Primer** |  |
| GAPDH | AACGACCACTTTGTCAAGC (forward) |
|  | TGAGGTCCACCACCCTGT (reverse) |
| SNX3 | GTCGTTGAAGGAGCTCTAGC (forward) |
|  | TTAAAAATAAACTTTTGTTG (reverse) |
|  |  |

**Table S1. Oligonucleotides used in research**
