# Supplementary material for: Integrating single-cell RNA-seq and bulk RNA-seq to construct prognostic signatures to explore the role of glutamine metabolism in breast cancer
Source: Front Endocrinol (Lausanne). 2023 Feb 10;14:1135297. doi: 10.3389/fendo.2023.1135297 (PMC9950399; doi:10.3389/fendo.2023.1135297)
Supplement: Supplementary file 3 [file DataSheet_1.docx]

Supplementary Material

# Supplementary Figures


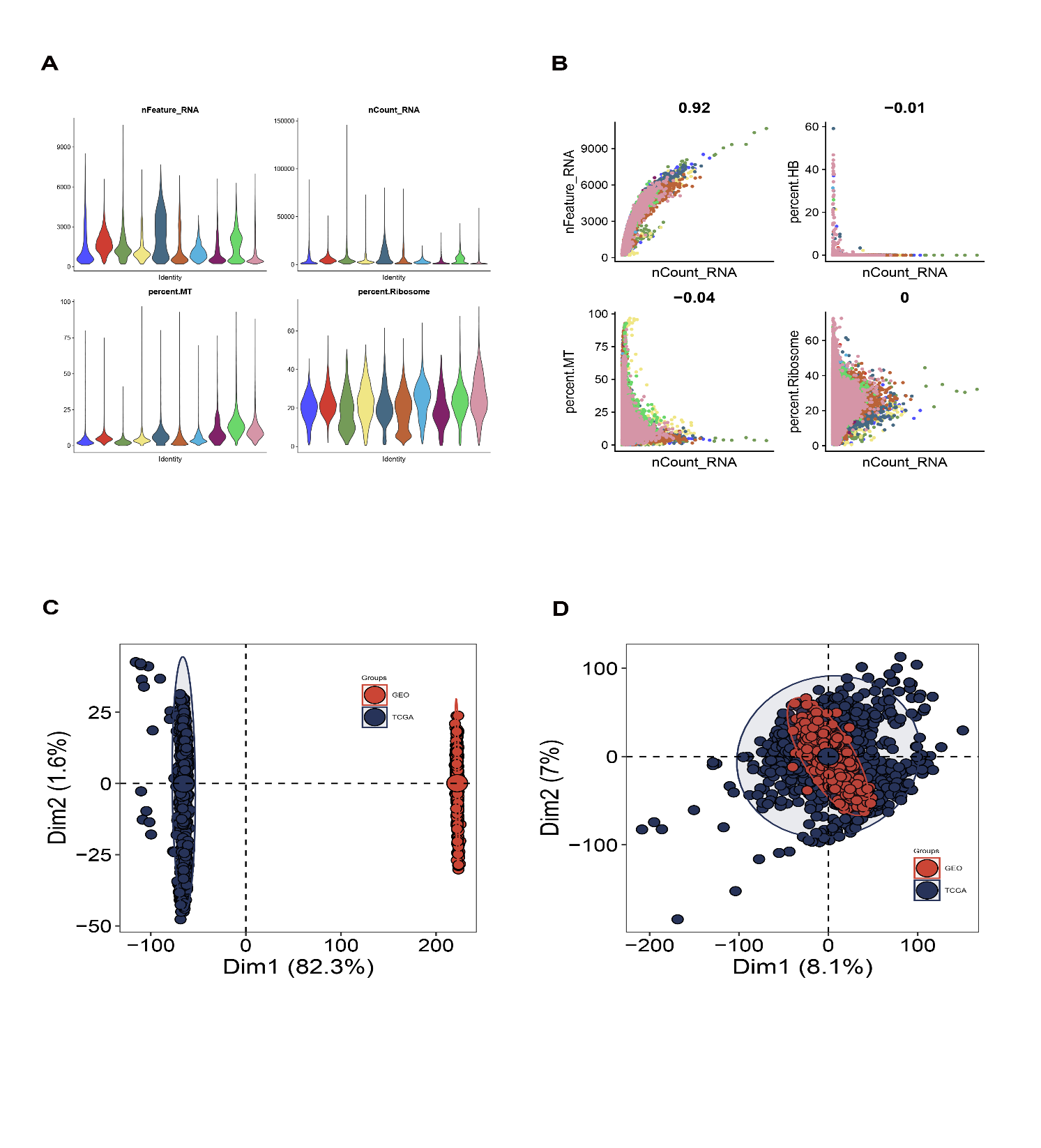


**Supplementary Figure S1.** Quality control and dimension reduction analysis of single-cell sequencing data. (A)By limiting the ratio of mitochondrial, ribosomal, and erythrocyte genes, cells that did not meet the criteria were removed. (B)Sequencing depth and total intracellular sequences exhibit significantly substantial positive associations (R=0.92). (C)No significant batch effects were observed in the TCGA cohort and GEO cohorts. (D)Removing the batch effect.
