# Supplementary figures and images for: Integrating single-cell RNA-seq and bulk RNA-seq to construct prognostic signatures to explore the role of glutamine metabolism in breast cancer
Source: Front Endocrinol (Lausanne). 2023 Feb 10;14:1135297. doi: 10.3389/fendo.2023.1135297 (PMC9950399; doi:10.3389/fendo.2023.1135297)

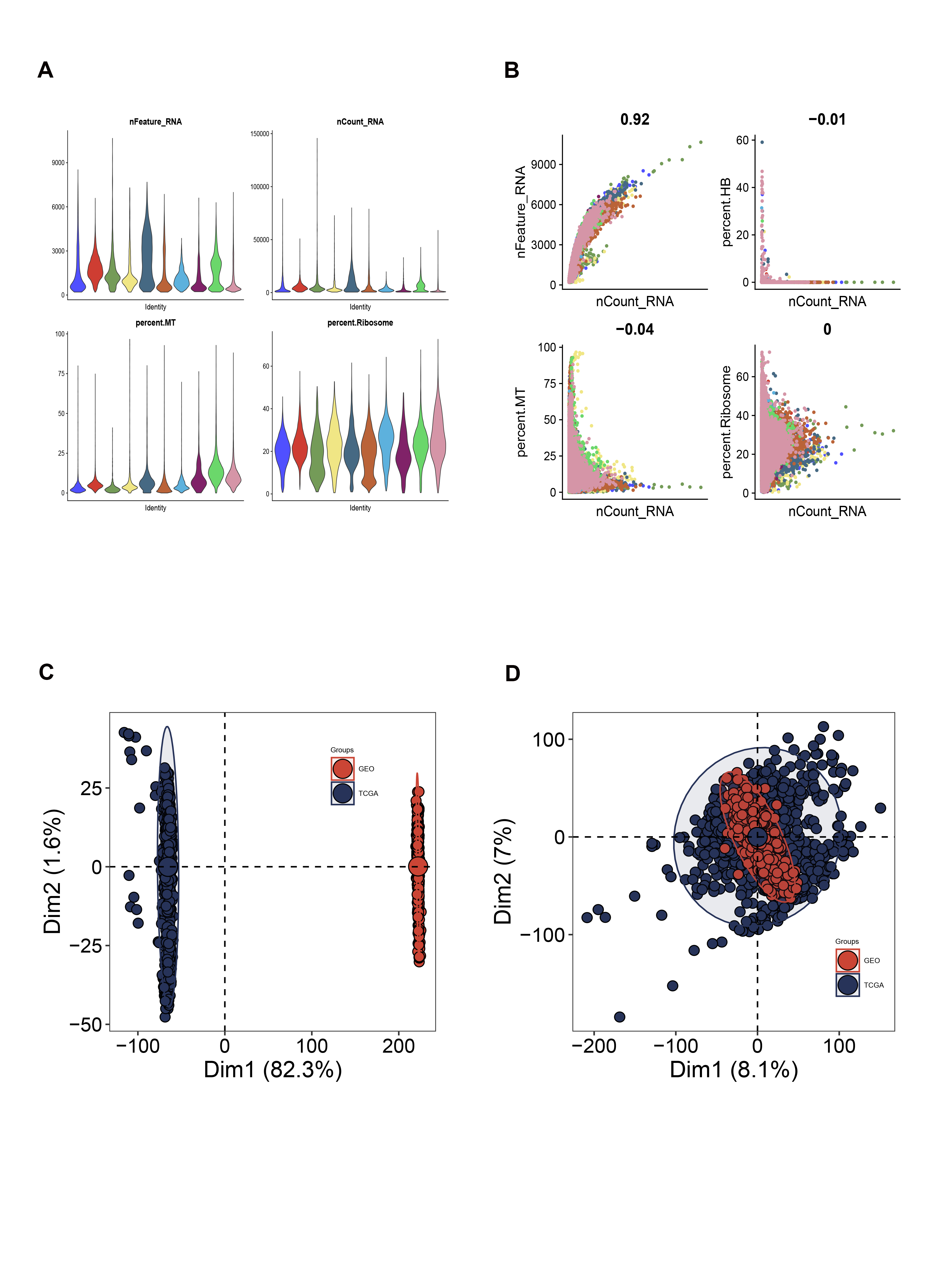

Supplement: Supplementary file 4 [file Image_1.tif]
